# Management of a Dutch resident barnacle goose *Branta leucopsis* population: How can results from counts, ringing and hunting bag statistics be reconciled?

**DOI:** 10.1007/s13280-017-0900-3

**Published:** 2017-02-18

**Authors:** Henk P. van der Jeugd, Anne Kwak

**Affiliations:** 1 Vogeltrekstation-Dutch Centre for Avian Migration and Demography, NIOO-KNAW, PO Box 50, 6700 AB Wageningen, The Netherlands; 2 Department of Animal Ecology and Ecophysiology, Institute for Water and Wetland Research, Radboud University, PO Box 9010, 6500 GL Nijmegen, The Netherlands

**Keywords:** Derogation shooting, Geese, Hunting bag, Management, Population model, Survival

## Abstract

The resident Dutch Northern Delta barnacle geese *Branta leucopsis* population expanded steadily since birds first bred in 1982, increasing agricultural conflict. Derogation shooting has been used since 2005 to scare geese from sensitive crops and to reduce population size. Numbers almost doubled to ca. 28 000 individuals during 2007–2014, despite annual removal of 15–25% of geese and adult and juvenile survival rates of 79 and 67% (cf. natural survival of 96% for both age classes). Simple population modelling, using estimated annual survival values and fixed fecundity, predicted a moderate increase to 21 500 individuals in 2014. It is unclear whether current harvest levels are sufficient to halt population growth. Shooting may be less effective because of the disproportionate take of immature post-breeding and of individuals from other populations in winter. Discrepancies between counted and modelled abundance call for caution and improved effect monitoring of derogation shooting before harvest levels are increased further.

**Electronic supplementary material:**

The online version of this article (doi:10.1007/s13280-017-0900-3) contains supplementary material, which is available to authorized users.

## Introduction

Many species of wild geese have increased in numbers over recent decades as a result of changing agricultural practices and reduced harvest levels. At the same time, ranges of both wintering as well as breeding birds have increased or shifted (van Eerden et al. 2005). This has led to a marked increase in the number of conflicts with agricultural interests. The barnacle goose is one such species, with birds wintering in continental Europe having adapted rapidly to a wide range of habitats in the temperate zone, thereby considerably shortening its migration route, with some individuals even adopting a sedentary life style (van der Jeugd et al. 2009). In the Netherlands, the species became established as a breeding bird in 1982 (Meininger and van Swelm 1994; Ouweneel 2001; Lensink et al. 2013), and the Dutch-breeding population has since grown to an estimated 13 800 breeding pairs by 2012 (Schekkerman 2012). The Northern Delta area is traditionally the stronghold of the Dutch population, holding ca. 50% of all breeding pairs (Feige et al. 2008; Boele et al. 2015). The amount of damage to agricultural crops and grasslands by barnacle geese has increased in the area. Compensation to farmers for crops assessed as being damaged by geese is provided by the Dutch ‘Fauna Fund’ (earlier Game Fund), who reimburse for yield losses on arable crops, the first cut of grass for silage, competition with livestock grazing and the effects of puddling during wet weather (van Roomen and Madsen 1992), but only after farmers have tried to scare geese from their land. Since 2005, derogation shooting of barnacle geese in the area has been permitted under license as a last resort. The annual hunting bag (hb) resulting from derogation shooting in the Northern Delta area increased from 377 barnacle geese in 2005 to 5324 in 2014 (data obtained from Faunabeheereenheid Zuid-Holland). There are no bag limits. From 2007 onwards, the population size of all summering geese in the area has been estimated by a single count conducted annually in July. A colour-ringing scheme has been established in this area since 2004, with a total of 1109 geese being colour-ringed up to and including 2014, with an additional 313 geese receiving metal rings only.

In this paper, we evaluate the effect of the derogation shooting on the local barnacle goose population by analysing the annual counts, hb statistics and demographic parameters based on re-sightings of colour-ringed geese. More specifically, we will establish whether counts of the local population size match projections of the population based on demographic data, whether bag statistics are in accordance with demographic data and counts, and how effective derogation shooting is limiting the local population.

## Materials and methods

### Counts

Counts of all summering geese in the entire Delta area in the provinces of Zeeland, Noord-Brabant and Zuid-Holland (including the Northern Delta area, which is part of the province of Zuid-Holland) were performed during mid-July in 2006 and 2007 by a small team of professionals from Sovon, the organisation responsible for coordinating bird counts across the Netherlands (van der Jeugd and Boer 2006; de Boer and van der Jeugd 2007). All geese in this area were counted on three consecutive days. From 2007 onwards, annual counts of all summering geese across Zuid-Holland, including the Northern Delta area, were also made by volunteers and nature reserve wardens, coordinated by Centrum voor Landbouw en Milieu (CLM), an independent consultancy working in the field of sustainable food, farming and rural development (Tolkamp and Guldemond 2007, 2008, 2009; Visser et al. 2010; den Hollander and Visser 2011, 2012; Keuper and Visser 2013, 2014). These counts were always performed on a single weekend in the middle of July. Tolkamp and Guldemond (2007) reported a number of problems with the first counts (e.g., areas counted twice, overestimates, ill-defined and overlapping counting areas, areas not covered), but in the course of the study the quality has improved, although coverage has decreased in recent years with many missing or incompletely counted areas. Numbers recorded in the annual CLM reports were aggregated into larger units (count areas) for the analyses presented here. The CLM reports did not aim to reconstruct actual numbers by taking missing values into account, or to undertake trend analyses. Numerical development of the population therefore was not known, and to reconstruct the population trend of barnacle geese in the Northern Delta area we developed a simple chain index, based on the areas counted in successive years. This was carried out by summing the counts in all areas that were counted in two consecutive years, and then calculating the % change from year one to the next based on these counts. The numbers that could be included in the calculation of the chain index varied between 41 and 100% of the total number counted in each year (average 76%).

**Fig. 1:**
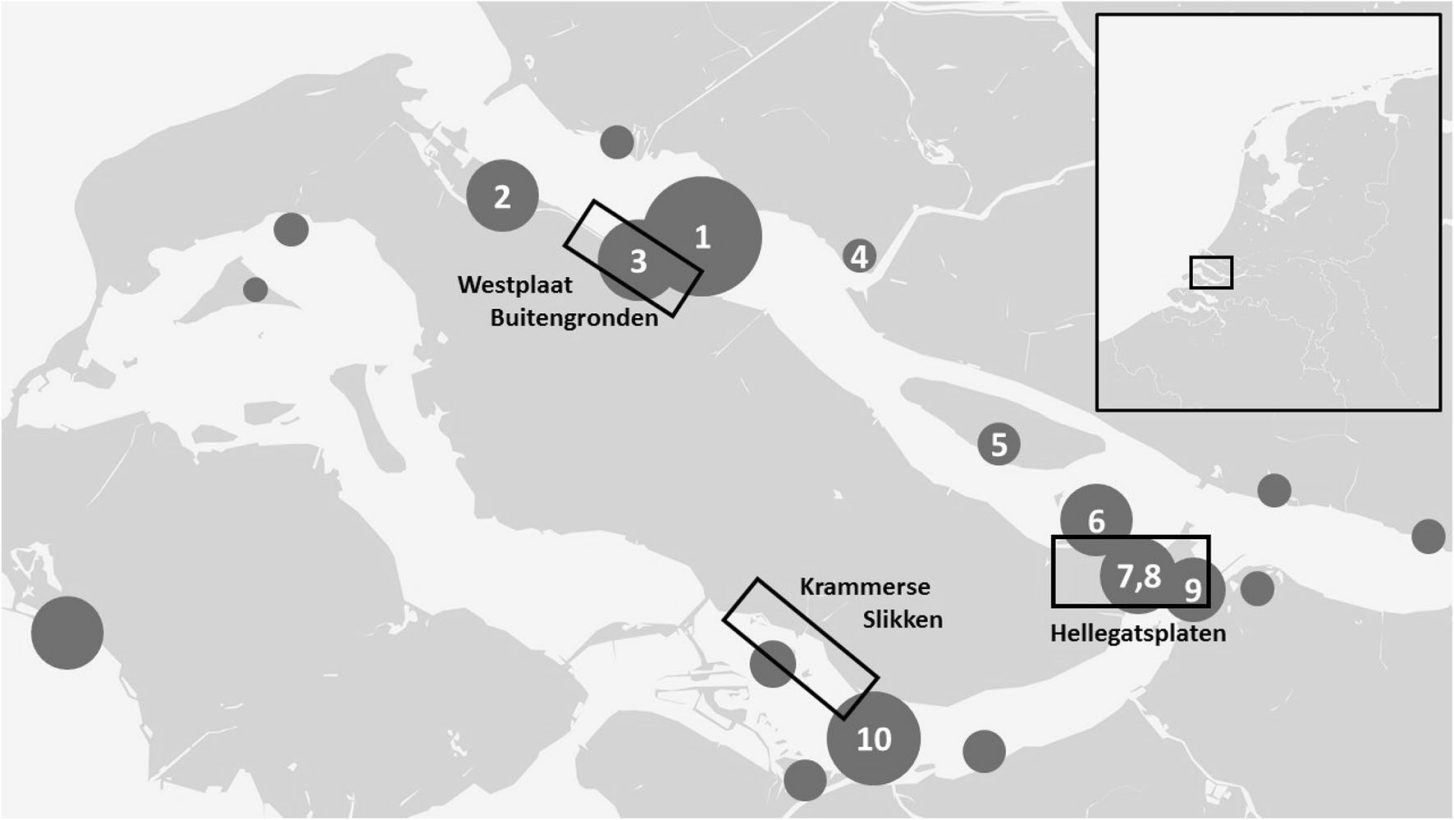
Map of the Northern Delta area in the southwest of the Netherlands, showing the three study areas where barnacle geese have been colour-ringed. *Dark grey circles* indicate the locations of barnacle goose colonies. The size of the *circles* is relative to the number of nests. Colonies where the number of nests is monitored are numbered *1*–*10*

In addition to the annual summer counts, many of the larger barnacle goose colonies in the Northern Delta area (Fig. 1) were visited annually during the breeding season to count the number of nests (Table S1). Nest counts are a relatively easy way to estimate the number of breeding pairs in colonial species. Nest counts in 10 colonies (Fig. 1) were made from 2004 through 2015 by the authors, a large number of volunteers and by personnel from Delta project management. In years where nest counts from a single colony were missing, the number was interpolated based on the number counted in the other years. Data are summarised by van der Jeugd and Kwak (2015) and used with permission from the various data owners for the analyses presented here.

### Hunting bag statistics

The number of shot birds in the Province of South Holland, which includes the Northern Delta area, was reported annually by local hunters to ‘Faunabeheereenheid Zuid-Holland’, and annual totals were made available for analyses. Derogation shooting under license of barnacle geese started in Zuid-Holland in 2005. There was no shooting in 2006, but from 2007 onwards barnacle geese were hunted annually, and the number of shot birds has risen steadily. No bag limits were imposed at any time. Initially, shooting was allowed from May through September. In 2012, the hunting season was extended and shooting was allowed from March through October. Most of the derogation shooting of barnacle geese in Zuid-Holland (55–75%) takes place in the Northern Delta Area, where most of the resident barnacle geese are found. The number of shot birds varies considerably by month. Most birds are shot in August and September, i.e., after the breeding season, when the population is augmented by young, fledged birds born that year.

As part of the derogation process, hunters are obliged to report ring numbers of shot geese to the Dutch ringing scheme (Vogeltrekstation NIOO-KNAW). Reports of shot geese with leg rings are important for survival analyses because they permit calculation of additional mortality in the population attributable to hunting. Up to and including 2014, 181 (12.7%) of the 1422 birds that were (colour-) ringed in the area were reported as having been shot. The reports have been used to calculate hunting mortality, to evaluate differences between sex and age classes in the probability of being shot, and to evaluate the extent to which derogation shooting targets the local breeding population. For the latter, we restricted analyses to the years 2012–2014, during which there was a longer hunting season (from March–October, rather than May–September), and there were more ringed birds reported as being shot in the Northern Delta area (2005–2011: 41 birds, 2012–2014: 108 birds).

### Ringing and re-sighting data

Ringing and ring re-sighting data for 1109 barnacle geese caught and colour-marked as breeding birds in the Netherlands from 2004 to 2013 inclusive were used in the analyses. Birds were rounded up during the annual moult in July at Hellegatsplaten (at the east end of Goeree-Overflakkee Island, Delta area, 51°42′N, 4°22′E) in 2004 and 2005, at Krammerse Slikken (in the southeast of Goeree-Overflakkee Island, Delta area, 51°40′N, 4°14′E) in 2007, 2008 and 2010 and at Westplaat Buitengronden (in the north of Goeree-Overflakkee Island, Delta area, 51°47′N, 4°08′E) in 2012 and 2013 (Fig. 1). In addition to the 1109 birds colour-ringed during the study, 313 individuals were marked with metal rings only. Young birds, varying in age between 4 and 8 weeks, and adults colour-ringed in approximately equal numbers were 545 and 564, respectively. In addition, 18 adult and 295 young birds were ringed with metal rings only. All birds were sexed by cloacal inspection (706 males, 674 females, 42 unknown).

Observations of colour-marked barnacle geese were used to calculate annual survival of barnacle geese in Northern Delta area. All the observations that were entered via the www.geese.org portal or otherwise added to the geese.org database, and which matched the ringing data of one of the colour-marked individuals were checked and assessed for inclusion in the analysis. All observations made before the bird’s ringing date were removed and, because the re-sighting frequency was high, with most of the birds being observed multiple times each year, single observations made more than two years after the preceding observation were scrutinised more closely. Most of these observations were deleted, because they were usually made by the same observers, by inexperienced observers, or at locations where the individual geese had never been seen before. When more than three observations were made at least 2 years after the preceding observation, or when re-sighting frequency was low throughout the whole life-history of the individual, these were maintained. After data cleaning, 23 189 observations remained for analyses. The mean number of observations per individual was 21 records (s.d. = 21, range = 1–112), and the median observation frequency was 14 sightings.

### Ring recoveries and survival analysis

To construct capture histories for the survival analyses based on ringing data, we grouped all re-sightings according to calendar years. Although this creates a long re-sighting period relative to the time interval over which survival is estimated, we follow O’Brien et al. (2005) who, from simulations using real data, recommended this approach on the basis that it makes best use of the data available, yields the highest re-sighting probabilities, thus increases precision of the estimates, and does not seem to give biased results. The median observation date per individual per year varied from 23 May to 13 July. Annual survival therefore is calculated from May–July in one year to May–July the in next year.

We used the Burnham capture-mark–re-sighting model (1993) to estimate annual survival. This model simultaneously uses re-sightings of live birds and recoveries of dead (shot) birds, making it possible to estimate four parameters: survival (*S*), which is the probability that an individual goose survives from 1 year to the next, reporting rate (*r*), which is the probability that a dead (shot) goose is being found and its ring number reported, re-sighting rate (*P*), which is the probability that an individual goose that is alive is being observed and reported and the fidelity parameter (*F*), which is the probability that an individual remains in the study area and retains its marks. Parameters were estimated using RMark (Laake 2013). In RMark, we tested models where parameter values depended on the covariates age (juvenile or adult) and time (year), both additives as well as in interaction with each other. We used the quasi AIC score to evaluate model parsimony. Quasi AIC combines model fit (the degree to which the model is able to represent the data) and model simplicity (the number of parameters the model contains). We calculated *c*-hat to investigate model fit. *C*-hat was 4.46 for the best model, indicating some lack of fit, and was used to adjust the qAIC values.

A total of 625 models were tested, investigating effects of time and age on all four parameters in all the possible combinations. The 10 best models always contained effects of time (year) for *S* and *P*, but for *r* and *F*, both models with and without a time effect were included (Table S2). Models, where *r* was constant, time-dependent or age-dependent (models 1–3), performed equally well. Because we were interested in time variation in *r*, and in age effects on survival, we used parameter estimates from model 4 for further analyses (Table S2, model 4; *S*_*t*+*a*_ *p*_*t*_ *r*_*ta*_ *F*_*t*+*a*_), which was only three AIC points away from the best model.

### Relating survival probabilities to counts and hunting bag statistics

**Fig. 2:**
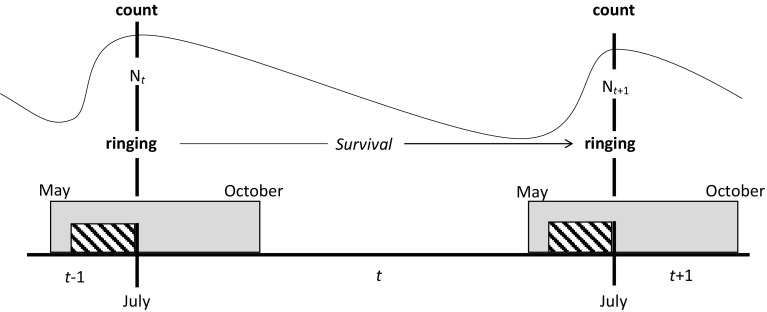
Time line showing the temporal distribution of evaluation events in relation to the barnacle goose annual cycle in the Northern Delta area. *Grey*-*shaded area* represents the hunting season, *hatched boxes* denotes the nesting period and nest counts

When relating survival probabilities to counts and hb statistics, we took into account that counts, ringing and the hunting period are not in phase with each other (Fig. 2). Each year, ringing took place in the week just prior to the mid-July count, during the annual moult at the end of the breeding season. Annual survival probabilities span the time period between May–July in 1 year and May–July in the next (see above), and thus are almost in phase with the counts. Reproduction takes place before ringing and counting, and fledged young of the year are included in ringing and in the annual count. The hunting season, however, starts in May (March) and runs through September (October), and on average exactly half of the birds are shot before ringing and the annual count, and half of the birds are shot after these events. To express the population size in the current year *N*_*t*+1_ as a function of the population size in the preceding year *N*_*t*_, the second half of the hunting bag hb_*t*_ in year *t* needs to be subtracted from *N*_*t*_. The remaining number is subject to natural mortality and, hence, has to be multiplied by the natural annual survival *S*_nat_ (i.e., survival not including hunting mortality; how *S*_nat_ can be inferred from the estimates of *S* is explained below). This gives the remaining population at the start of the next breeding season in year *t* + 1. The new recruits from reproduction *R*_*t*+1_ during that breeding season are then added to that number, and the first half of the hunting bag hb_*t*+1_ in year *t* + 1 is subtracted to give the population size that is counted in year *t* + 1.

1 $$ N_{t + 1} = \left( {\left( {N_{t} -0.5 \cdot {\text{hb}}_{t} } \right)\cdot S_{\text{nat}} } \right) + R_{t + 1} - 0.5 \cdot {\text{hb}}_{t + 1} . $$

Using Eq. (1), it is easy to verify whether hb statistics and counts are in accordance with each other by comparing calculated and counted values of *N*_*t*+1_. It is also possible to estimate hunting mortality from the number of ringed birds reported shot. This number gives an estimate of the true number of ringed birds shot when it is divided by the reporting rate *r*. The estimate of the true number of ringed birds shot divided by the number of birds ‘at risk’ (calculated as the expected number of colour-marked birds alive during each summer using the annual survival estimates) yields an estimate of hunting mortality. Annual numbers ‘at risk’ are calculated from the number of barnacle geese ringed in each year, multiplied by the appropriate annual survival probabilities (van der Jeugd 2013). When hunting mortality is related to annual survival from the survival modelling, the *y*-intercept of the relationship, where hunting mortality is zero, gives an estimate of the natural survival without hunting, *S*_nat_.

### Population model

To forecast the population size from its baseline value in 2007, we assumed a constant fecundity *F* of 0.2 females per female (0.4 fledglings per pair). Fecundity was based on counts of the total number of fledged young in brood-rearing areas in July, divided by the total number of adult birds in the same brood-rearing area. In total, data from five brood-rearing areas were available in the period 2004–2007, but recent information is lacking (van der Jeugd 2012, 2013). Variation between brood-rearing areas was considerable, but annual variation was relatively small (2004: 0.28, 2005: 0.55, 2006: 0.38, 2007: 0.36). Sample sizes varied between 1045 and 4057 breeding pairs. We used a simple population model where the numbers in each year are calculated from the numbers in the preceding year, according to the annual survival estimates for juvenile and adult birds obtained from the survival modelling:

2 $$ N_{t + 1} = \left( {\left( {N_{t} /(1 \, + \, F)} \right) \cdot Sa_{t \to t + 1} + \left( {\left( {N_{t} /(1 + F)} \right) \cdot F} \right) \cdot Sj_{t \to t + 1} } \right) \cdot 1 + F, $$

where *N*_*t*_/(1 + *F*) calculates the adult part of the population in year *t* and ((*N*_*t*_/(1 + *F*))·*F*) calculates the juvenile part of the population in year *t*. The adult and juvenile parts of the population are multiplied by their respective annual survival, and finally, the remaining total of both parts in year *t* + 1 is multiplied by 1 + *F* to add the juveniles produced in year *t* + 1. Note that *F* here is calculated from counts of all juvenile and adult birds in July, and, hence, is the average for all age classes including 1-year-old birds (following Lee et al. 2016). Because our estimate of *F* is based on juvenile counts in the period 2004–2007 only, we also projected numbers using values for *F* of 0.15 and 0.25 females per female, to account for possible changes in fecundity. This creates an upper and a lower population trajectory between which we assume the actual population size should be. The forecasted population size is then compared to the actual counts.

## Results

### Reconstructing counts

In July 2007, 13 223 and 14 995 barnacle geese were counted in the Northern Delta area by Sovon and CLM, respectively, with only 1 week apart. Although numbers deviated, we consider the CLM count in 2007 as our baseline population estimate since CLM coordinated all subsequent counts. Between 2004 and 2014, the index showed an increase of the population of 89% compared to the level in 2007, to 28 316 geese.

Nest counts in ten colonies in the Northern Delta area (Fig. 1) confirm an increase in the number of the barnacle geese breeding in the area; the number of nests in these colonies increased from 2097 in 2004 to 2931 in 2007 and 4676 in 2009. There was no further increase in the total number of nests recorded after 2009, although there was substantial variation between colonies in their numerical development. Counts made in 2014 found 4192 nests in the study area, an increase of 43% over 2007.

### Hunting bag statistics

**Fig. 3:**
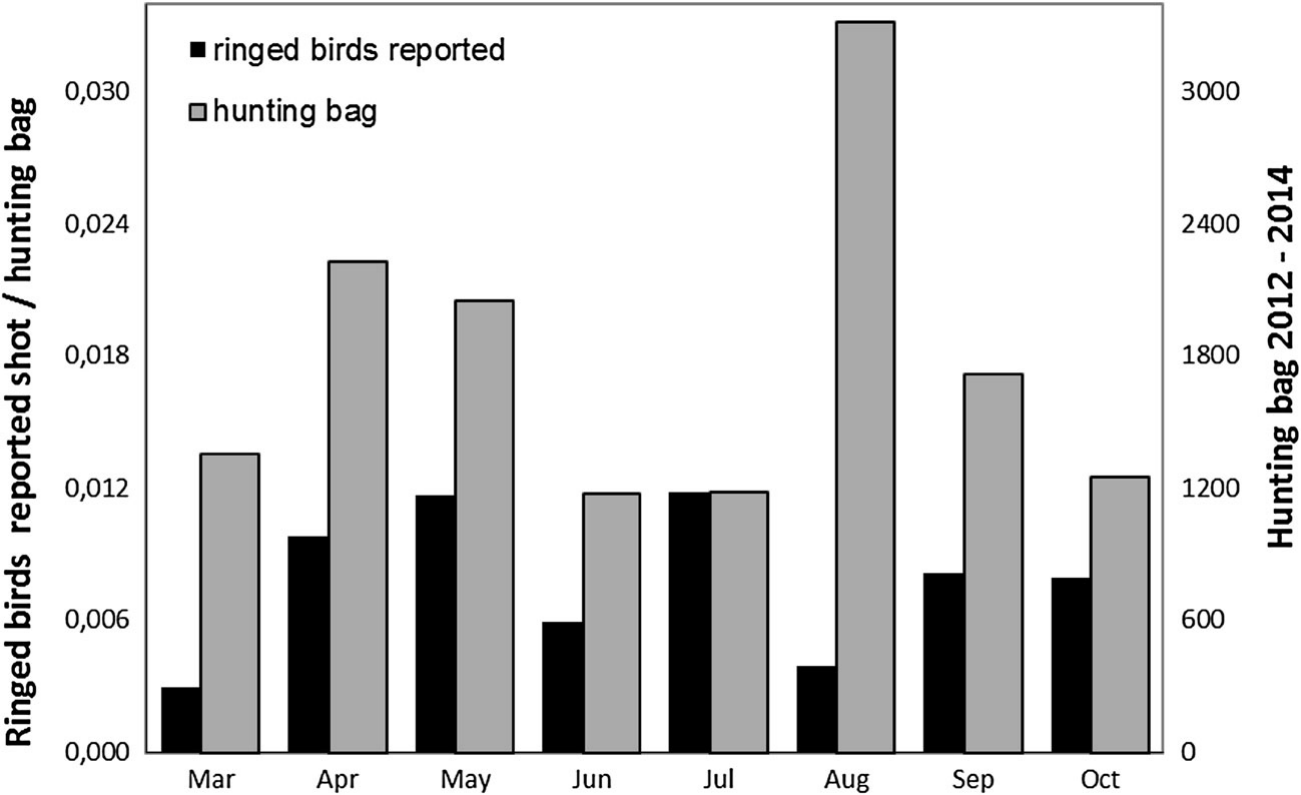
Barnacle goose hunting bag in the Northern Delta area broken down by month (*grey bars right hand y*-axis), and the number of ringed barnacle geese reported shot over the total hunting bag in that month (*black bars left hand y*-axis) in the period 2012–2014

Of the 1422 barnacle geese ringed in the Northern Delta area, 181 (12.7%) were reported shot by the end of 2014: 149 in the Northern Delta area and 32 elsewhere. Birds shot elsewhere were recovered predominantly in the neighbouring provinces of Noord-Brabant (13) and Zeeland (4), and elsewhere in Zuid-Holland (6). The remaining nine were shot in other parts of the Netherlands (7), in Belgium (1) and Estonia (1). Almost one quarter of all shot barnacle geese are shot in August (Fig. 3). The number of ringed shot barnacle geese that is reported, divided by the total number of barnacle geese shot, provides an index of the extent to which the derogation shooting targets the local breeding population. We restricted this analysis to the years 2012–2014, during which the hunting season was extended to March–October, and 108 birds were reported shot out of the 149 birds that were reported as being shot in the Northern Delta area in total.

The proportion of locally ringed birds in the monthly barnacle goose hbs was the lowest in March and August, indicating that shooting is least effective in these months with regard to targeting the local breeding population, but there was little variation among the other months (Fig. 3). At the same time, most birds (23% of the total) were shot in August. No foreign-ringed barnacle geese were reported shot in the Northern Delta area, and only two birds reported shot were ringed during winter in the Netherlands outside the area. As the birds were shot in July and September, it is unlikely that they were wintering birds from the Arctic population.

There was a slight difference in the probability of being shot and reported between barnacle geese ringed as juvenile and birds ringed as adult (ringed as juvenile 11.4%, ringed as adult 14.4%, *χ* ^2^ = 2.74, *P* < 0.1). However, since birds ringed as juveniles are less likely to remain in the area (van der Jeugd 2013), they are therefore also less prone to be shot during their life. Indeed, when the analyses are restricted to the first year after ringing only, the difference disappears (ringed as juvenile 4.9%, ringed as adult 4.4%, *χ* ^2^ = 0.42, *P* = 0.42). Among adults, males tended to be shot and reported somewhat more often than females (male 17.0%, female 12.1%, *χ* ^2^ = 2.72, *P* = 0.10).

### Annual survival

**Fig. 4:**
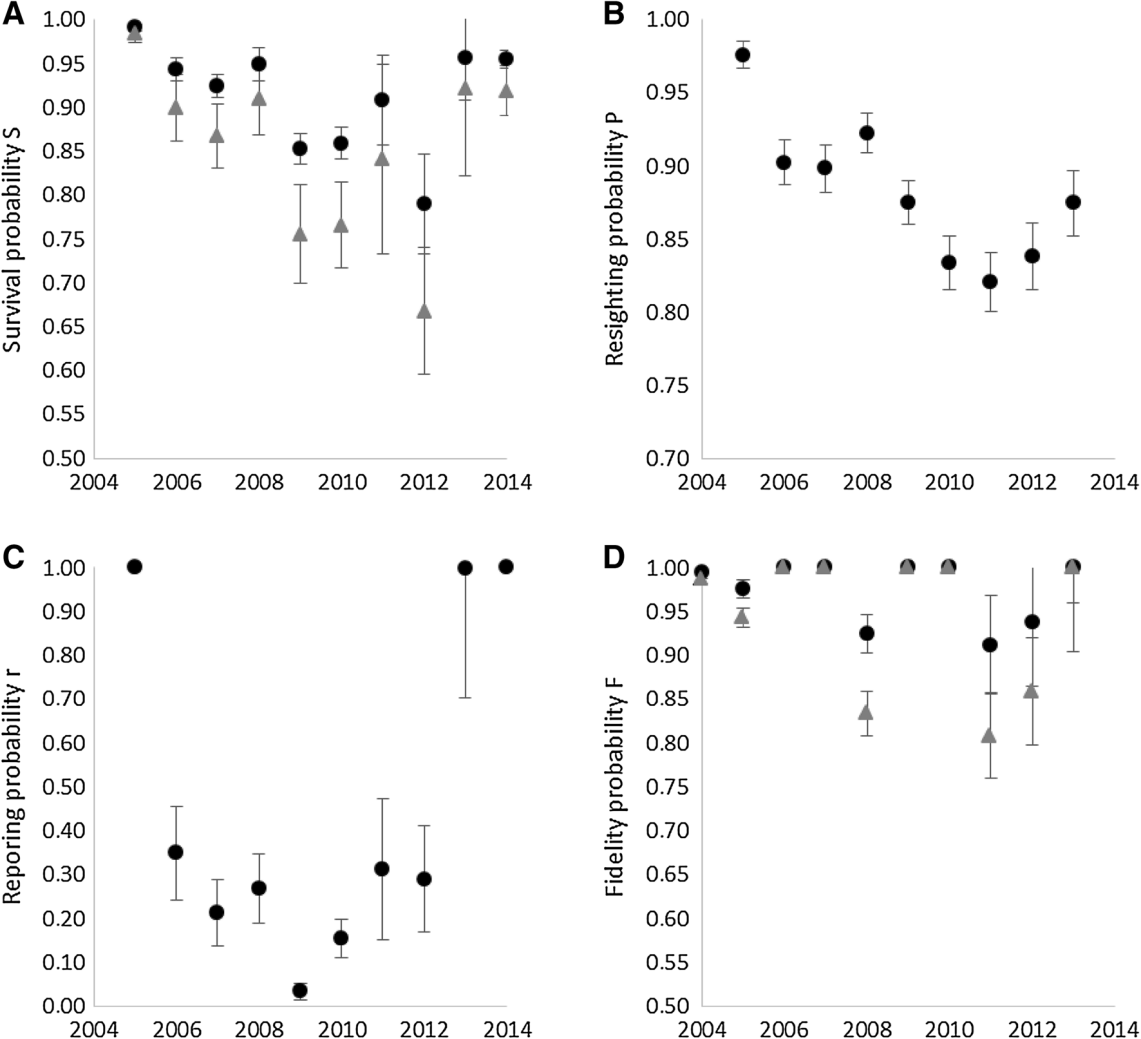
Annual survival probability *S* (panel **A**), re-sighting probability *P* (panel **B**), reporting probability *r* (panel **C**), and fidelity probability *F* (panel **D**), for barnacle geese ringed in the Dutch Northern Delta area, based on ring re-sightings and recoveries. Parameter estimates are taken from model *S*_time+age_ *p*_time_ *r*_time_ *F*_time+age_ (Table S2). The parameters *S*, *r* and *F* are estimated separately for adults (*black dots*) and juveniles (*grey triangles*)

Annual survival of barnacle geese ringed in the Dutch Delta area was higher in adults than in juvenile birds during their first year of life. Initially, survival was very high in both age classes, with birds ringed in 2004 and 2005 experiencing an annual survival probability of 0.98. Survival decreased markedly between 2005 and 2009. Survival was the lowest in 2009–2012. In those years, adult survival varied between 85 and 91%, and juvenile survival ranged from 67 to 76%. Survival for both age classes increased somewhat again in 2013 and 2014 (Fig. 4a).

The re-sighting probability was initially high, with no difference between the age classes, but decreased during the study period (Fig. 4b). Reporting rate first declined each year up to 2009/2010, then increased during the second half of study to more than 50% in adults and 37% in juveniles (Fig. 4c). Note that the values for the first and last year should be treated with care as these were both estimated as exactly one by the model. The Fidelity parameter was high throughout the study period (Fig. 4d). We used the survival and reporting rate parameter values (taking into account the timeline in Fig. 2), to analyse whether data from the counts, hb statistics and survival modelling are in accordance with each other.

### Relating survival probabilities to counts and hunting bag statistics

**Fig. 5:**
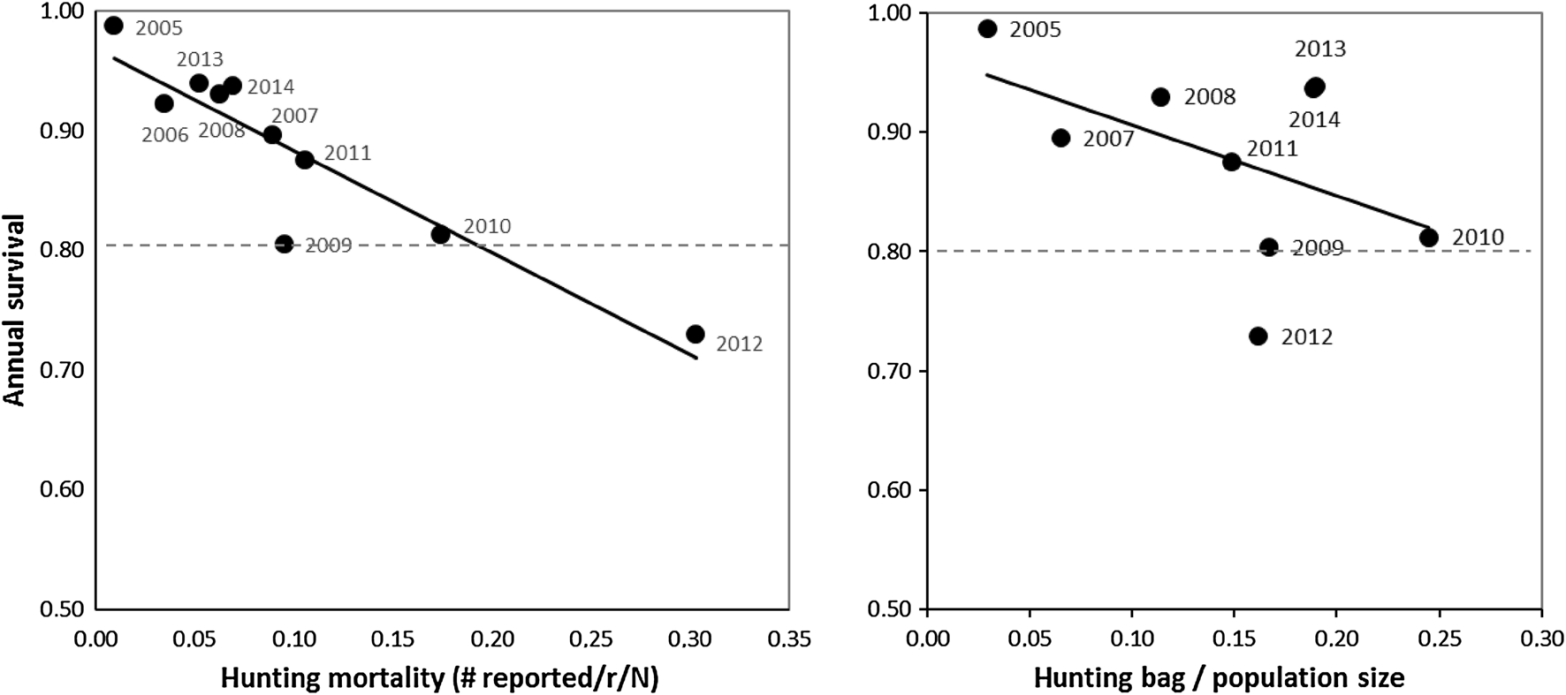
*Left panel* hunting mortality, calculated as the number of ringed barnacle geese reported shot, divided by the reporting rate *r* and the number of ringed birds alive (birds ‘at risk’) is negatively related to annual survival in a population of barnacle geese in the Northern Delta area. *Right panel* relationship between the hunting bag, expressed as a proportion of the total population (derived from an index based on annual counts), in relation to annual survival. *Dotted lines* represent the annual survival level below which the population should decline (see text)

First, we related the hunting mortality calculated from the reporting rate *r* and the actual numbers of birds reported in each year to the annual survival probabilities (Fig. 5a). There appeared to be a close relationship between the annual survival probability and our estimate of hunting mortality, the only exception being in 2009 when low survival coincided with a hunting mortality of only 0.11, attributable to a very low number of shot ringed birds reported that year. The *y*-intercept of the relationship, which gives an estimate of the natural survival without hunting, is approximately 0.96. Hunting mortality was the highest between 2009 and 2012 (Fig. 5), but has decreased again in more recent years.

**Fig. 6:**
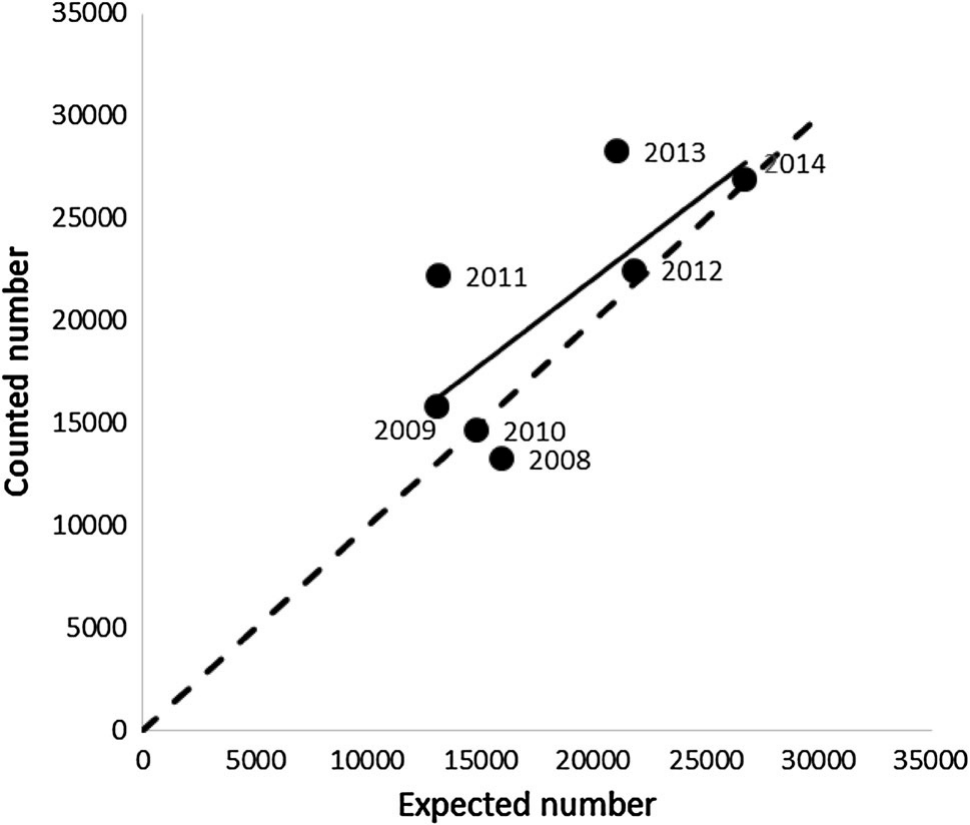
Numbers of barnacle geese counted in the Northern Delta area each year, corrected using a chain index (see text for details), in relation to the expected number, based on the number counted in the preceding year, fixed natural survival and reproduction, and the reported hunting bag (see text)

The annual July count was related to the hb using annual survival probabilities and a constant reproduction of 0.4 fledglings per pair using Eq. (1). For natural survival *S*_nat_, we took the value of 0.96 as estimated above. On average, the counted numbers were higher than expected, with relatively high counts in 2009, 2011 and 2013 (Fig. 6).

### Population model

**Fig. 7:**
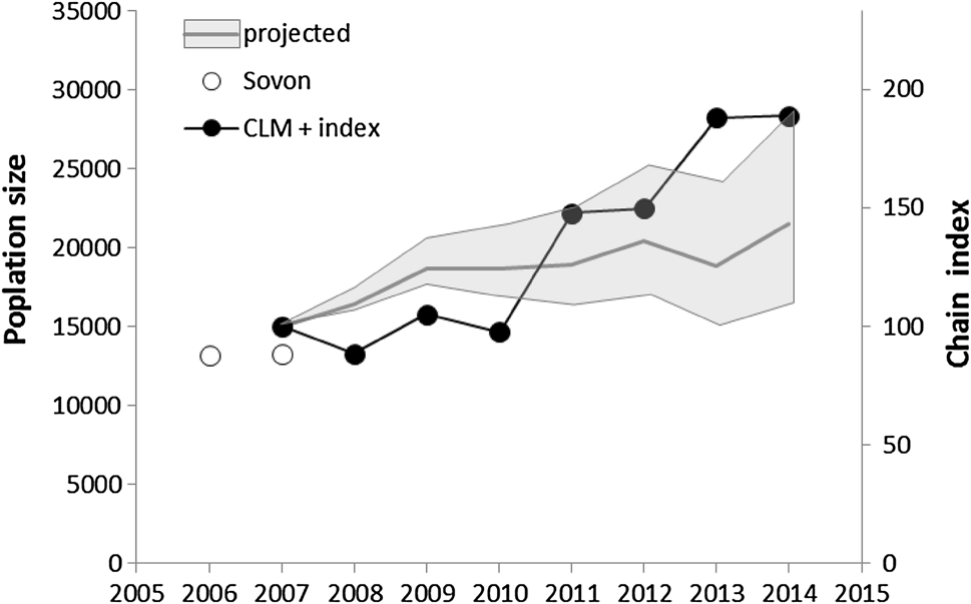
Counts (*filled* and *open circles*) and projected size (*shaded area*) of the barnacle goose population in the Northern Delta area. Population projection is based on a simple population model assuming survival varying by age and year and a constant fecundity of 0.2 females per female (see text), and lower and upper limits of 0.15 and 0.25, respectively

We projected the population size of barnacle geese in the Northern Delta area starting from the CLM count in 2007 using Eq. (2). We used the annual survival probabilities for juvenile and adult barnacle geese obtained from our survival modelling (see above) and a fecundity of 0.2 females per female based on juvenile counts in 2004–2007 (see “Methods” section) as well as 2.5 females per females as an upper limit, and a more conservative value of 0.15 as a lower limit (Fig. 7). The projection indicates a moderately growing population in most years except 2012 when it should have slightly decreased. Corrected counts indicate a population growth that deviates from the projection in some years, and is generally higher in the second half of the study period. The counted number in 2014 matches the upper limit of the projection (Fig. 7).

## Discussion

Since 2005, the resident population of barnacle geese in the Dutch Northern Delta area has been subject to derogation shooting as a measure to scare geese from sensitive crops and to reduce the population size. The size of the population has been monitored annually by means of a single count in July of each year. Although counts were incomplete in some years, there were signs of overestimation in others and as a result the numerical trend of the population has not been evaluated properly. A reconstruction of counts using a simple chain index indicated that the barnacle goose population of the Dutch Northern Delta area almost doubled in size between 2007 and 2014, despite derogation shooting with an annual hb that amounted from 15% to almost 25% of the total population size. Nest counts in ten large colonies confirmed the increase, but at a lower rate, with an increase of 43% between 2007 and 2014. The population model also indicated a slower growth rate than was borne out from the annual July counts, but nevertheless, derogation shooting has not resulted in a decline of the population, as was the aim.

An analysis of reports of shot ringed geese indicates that derogation shooting was not effective in August and March, albeit for different reasons. The March hunt likely also targets wintering Arctic barnacle geese instead of local breeders, hence the low proportion of ringed birds among the shot birds. There were no foreign-ringed birds among the ringed birds reported shot in this period. However, given the large size of the Russian Arctic population (ca. 1.2 million in 2014, K. Koffijberg, unpubl.) and the low proportion of geese bearing rings in relation to the numbers shot, the probability of encountering such rings is probably very low. The August hunt may not be effective in reducing the population size because of the large number of newly fledged young birds that have just entered the population. This lowers the proportion of ringed birds since ringed juveniles only result from ringing activities in the current year, if any, while older ringed birds in the population result from all ringing activities since the start of the study. For long-lived species, reducing the numbers of adult, breeding birds have the largest effect on the population growth. This means that, if the derogation shooting is primarily implemented to reduce the population size, hunting should be concentrated during late spring when adult breeding birds of the local population can be targeted. This is difficult, however, because birds already start nesting during this time and can only be shot during short foraging bouts.

Reporting rate of shot ringed birds first decreased but then increased substantially during the study period, indicating a growing willingness of local hunters to report shot ringed geese and an increasing awareness that these data can be used to evaluate the effect of derogation shooting. As was pointed out by Tombre et al. (2013), it is important to involve all stakeholders in adaptive goose management. In the Northern Delta area, an effort has been made to increase contacts between researchers, volunteer ring readers and the local hunting community, apparently with the desired effect.

We could not find any clear difference between birds ringed as juvenile and birds ringed as adults in the probability of being shot. Adults tend to be shot slightly more often, but this is most likely due to the fact that juveniles more often leave the area to breed elsewhere (van der Jeugd 2001, 2013), while adult birds remain in the study area and thus are at risk of being shot.

Annual survival was initially very high, owing to the fact that that the population did not suffer the burden of migration and hunting was still absent (van der Jeugd et al. 2009). Survival decreased when derogation hunting started, but has increased again during the most recent years. The most likely explanation for this increase is the fact that the hb has not kept up with the further population increase in those years (Fig. 5). Integrating survival, reproduction and hb, we evaluated the count data and conclude that in many years, counted numbers were higher than expected. There is no indication that hbs are incorrect.

There was a negative correlation between the calculated hunting mortality, based on the number of shot ringed geese reported and the annual reporting rates, and the annual survival probabilities. This suggests that hunting mortality is additive rather than compensatory in this population, as was also suggested in other studies of harvest rates in geese and is probably a general feature of long-lived species (e.g., Gauthier et al. 2001). The relationship clearly shows that hunting mortality was the highest in the period 2009–2012, but lower both before and after it. The relationship also yields a measure of the natural survival, without hunting, in this population which is approximately 0.96. This compares well to the observed survival during the first 2 years of the study, when derogation shooting had just started, and with the value of 0.97 reported by van der Jeugd et al. (2009).

There is also a negative, albeit weaker correlation between the hb expressed as a proportion of the population size (chain index-corrected numbers) and annual survival. According to this relationship, the annual hb should represent at least 20% of the local population to result in any reduction (Fig. 5). Because there are uncertainties in the actual population size estimates as well as in the fecundity and survival estimates, individual years deviate from the found relationship, but it is clear that only in 2009, 2010 and 2012 hbs in relation to the population size estimate were large enough and annual survival was low enough to realise a reduction of the population. These results are also corroborated by the population projection starting from the count in 2007. Based on an average fecundity of 0.2, the population increased moderately between 2007 and 2014 despite the derogation shooting, and only in 2009, 2010 and 2012, it did remain stable or decreased. There is, however, a large degree of uncertainty in the projection as fecundity levels have been based on data gathered in 2004–2007, and recent information on breeding success is lacking. Lowering fecundity from 0.2 to 0.15, for example, already yields a stable population.

Reducing thriving populations of geese by means of hunting is not easy. For example, to halt growth in the multi-million lesser snow geese *Chen caerulescens caerulescens* population in North America, a special continent-wide ‘conservation order hunt’ and a spring hunt in Canada was implemented to reduce adult survival through increased hunting mortality, which was judged to be the most cost-effective approach to reversing population growth. Despite a huge effort, harvest rates of lesser snow geese have not been high enough to decrease the population (Alisauskas et al. 2011). In Quebec, Canada, a spring hunt implemented in 1998 eventually succeeded in stabilising the greater snow geese *C. c. atlantica* population at between 700 000 and 1 000 000 birds (Reed and Calvert 2007), although the target of between 500 000 and 750 000 birds has not yet been met (Bélanger and Lefebvre 2006; Anonymous 2013). Interestingly, another temperate barnacle goose population on Gotland, Sweden that showed a similar increase to the population of the Northern Delta area (Larsson and Forslund 1994; Larsson and van der Jeugd 1998) has greatly declined in numbers in recent year after the establishment of white-tailed eagles (Larsson unpubl.). Also in the Netherlands, local predator dynamics can have profound effects on the development of barnacle goose colonies (Kleefstra 2010).

We conclude that current harvest levels in the barnacle goose population of the Northern Delta area, despite its relatively small size, seem insufficient to reverse population growth and that the effect of the derogation shooting is low, caused by a disproportionate number of immature individuals just after the breeding season, and by shooting individuals that are not belonging to the target population during late winter. In order to resort maximum effect, derogation shooting should be directed towards adult, reproducing breeding birds at the start of the breeding season.

However, caution is needed before harvest levels are increased further since there is considerable uncertainty about the exact population change since the derogation started. The July count indicates a much larger population increase than the population projection does, and only matches the very upper limit of the projection. Moreover, nest counts show a more moderate increase that is more in line with the population projection. It is possible that the July population is augmented by individuals from elsewhere, i.e., that are not part of the local breeding population. This, however, is difficult to assess as there are no ringing activities in other breeding colonies of barnacle geese in the Netherlands.

Monitoring of the effect of the derogation shooting by means of a single July count of the entire population is potentially possible, but only when strictly coordinated and with sufficient coverage. Proper trend analysis should be performed on an annual basis in order to be able to immediately act on changes in the population trend during the subsequent hunting season, either by increasing or decreasing effort. Additional demographic monitoring through colour-ringing, juvenile counts and simple population modelling in addition to counting yields important information on hunting efficiency and can even replace counts altogether as long as parameters can be estimated with precision and without bias. To achieve such a form of adaptive management, it is important to identify, inform and involve all stakeholders (Tombre et al. 2013) in a constructive way.

## Electronic supplementary material

Below is the link to the electronic supplementary material.

Supplementary material 1 (PDF 530 kb)
