# Supplementary material for: Management of a Dutch resident barnacle goose Branta leucopsis population: How can results from counts, ringing and hunting bag statistics be reconciled?
Source: Ambio. 2017 Feb 18;46(Suppl 2):251–61. doi: 10.1007/s13280-017-0900-3 (PMC5316331; doi:10.1007/s13280-017-0900-3)
Supplement: Supplementary file 1 — Supplementary material 1 (PDF 530 kb) [file 13280_2017_900_MOESM1_ESM.pdf]

Ambio

Electronic Supplementary Material

This supplementary material has not been peer-reviewed

Title: **Management of a Dutch resident barnacle goose *Branta leucopsis* population: How can results from counts, ringing and hunting bag statistics be reconciled?**

Authors: Henk P. van der Jeugd, Anne Kwak

Table S1 Number of nests counted in ten barnacle goose colonies in the northern Delta area in 2004-2014, including the authority. Numbers in last column refer to remarks below the table.

**Colony name**	**2004**	**2005**	**2006**	**2007**	**2008**	**2009**	**2010**	**2011**	**2012**	**2013**	**2014**	**Authority**
Slijkplaat, Haringvliet	714	935	1447	1112	1288	1518	1593	1378	1330	988	1023	DPM
Scheelhoek eilanden	305	366	567	355	410	573	511	480	411	398	293	DPM
Westplaat Buitengronden	0	*(71)*	*(142)*	*(212)*	283	*(388)*	*(493)*	*(598)*	703	*(590)*	*(476)*	*authors*
Beeninger Slikken	23	33	16	13	14	*(18*	*(17)*	31	31	*(31*	*(31)*	*Kuijpers & van der Heiden*
Tiengemeten					0	*(25)*	*(49)*	74	*(106)*	*(139)*	171	Natuurmonumenten
eilanden Ventjagersplaten	10	76	67	130	246	224	367	298	360	591	575	DPM
Hellegatsplaten, Ooltgensplaateiland, lange eiland	146	176	250	239	236	236	274	166	209	192	143	DPM
Hellegatsplaten, other	372	360	347	378	*(373)*	*(373)*	*(433)*	*(263)*	*(331)*	*(304)*	*(226)*	*authors*
Krib Hellegat / Banaan	260	375	*(309)*	242	251	393	347	275	360	473	*(473)*	*DPM*
Noordplaat, Volkerrakmeer	267	252	*(251)*	250	512	928	514	560	757	780	*(780)*	*DPM*
**total excluding interpolated counts**	**2097**	**2573**	**2694**	**2719**	**3240**	**3872**	**3606**	**3262**	**4161**	**3422**	**2205**	
**total including interpolated counts**	**2097**	**2644**	**3395**	**2931**	**3613**	**4676**	**4599**	**4123**	**4598**	**4485**	**4192**	

1: Numbers in brackets interpolated

2: Numbers in brackets interpolated, last year(s) assumed the same as year before

3: Numbers assumed proportional to other part of this colony

4: Numbers in 2011-2014 are incomplete

Table S2 The ten best-fitting models for survival of barnacle geese in the Northern Delta area Netherlands. Models consider survival (S), resighting probability (P), reporting probability (r) and the fidelity parameter (F). Parameters are modelled as a function of time (year) and age. For further analyses, parameter estimates for S and r were chosen from model 4

	Model	npar	AICc	ΔAICc	Weging	Deviance
1	S(time),P(time)r(.),F(time + age)	33	7089,14	0,0000	0,2947	1265,38
2	S(time),P(time),r(age),F(time + age)	34	7089,41	0,2689	0,2576	1263,62
3	S(time),P(time),r(time),F(time + age)	43	7089,43	0,2880	0,2552	1245,34
4	S(time + age),P(time),r(time),F(time + age)	44	7092,46	3,3169	0,0561	1246,33
5	S(time),P(time),r(time + age),F(time + age)	44	7092,80	3,6631	0,0472	1246,67
6	S(time + age),P(time),r(time + age),F(time)	44	7095,12	5,9785	0,0148	1248,99
7	S(time + age),P(time),r(time),F(time)	43	7095,13	5,9945	0,0147	1251,04
8	S(time + age),P(time),r(time + age),F(.)	35	7095,98	6,8406	0,0096	1268,16
9	S(time + age),P(time),r(time),F(age)	35	7096,39	7,2500	0,0079	1268,57
10	S(time + age),P(time),r(time + age),F(~time + age)	45	7096,43	7,2940	0,0077	1248,26
